# Non-invasive pulse wave analysis for monitoring the cardiovascular effects of CO_2_ pneumoperitoneum during laparoscopic cholecystectomy- a prospective case-series study

**DOI:** 10.1186/1471-2253-14-98

**Published:** 2014-10-31

**Authors:** Péter Sárkány, Szabolcs Lengyel, Réka Nemes, Lívia Orosz, Dénes Páll, Csilla Molnár, Béla Fülesdi

**Affiliations:** Department of Anesthesiology and Intensive Care, University of Debrecen, Medical and Health Science Centre, Nagyerdei krt. 98, H-4032 Debrecen, Hungary; 1st Department of Medicine, University of Debrecen, Medical and Health Science Centre, Nagyerdei krt. 98, H-4032 Debrecen, Hungary

**Keywords:** Laparoscopic cholecystectomy, Hemodynamic changes, Applanation tonometry

## Abstract

**Background:**

Peritoneal insufflation results in hemodynamic changes during laparoscopic cholecystectomy. The aim of the present work is to test whether non-invasive applanation tonometry is suitable for reflecting these hemodynamic alterations.

**Methods:**

41 patients undergoing laparoscopic cholecystectomies were monitored using the SphygmoCor pulse wave analysing system. Peripheral blood pressures (PBP), central aortic blood pressures (CBP), augmentation index (ALX@HR75) and subendocardial viability ratio (SVR) were measured at rest (Phase 1), after anesthetic induction (Phase 2), after peritoneal inflation (Phase 3) and after peritoneal deflation (Phase 4).

**Results:**

Induction of anesthesia resulted in a statistically significant reduction in both the peripheral blood pressure and central aortic pressures, accompanied by a decrease in augmentation pressure and augmentation index. Peripheral blood pressures did not change along with the peritoneal cavity insufflation, except for a moderate increase in systolic blood pressure. In contrast to this, an increase could be observed in central aortic pressure (106.77 ± 18.78 vs. 118.05 ± 19.85 mmHg, P < 0.01) which was accompanied by increased augementation pressure (18.97 ± 10.80 vs. 31.55 ± 12.01; P < 0.001) and augmentation index (7.31 ± 5.59 vs. 12.61 ± 7.56, P < 0.001), indicating a rise in peripheral arterial stiffness.

**Conclusions:**

The Sphigmocor pulse wave analysis system can be reliably used for detecting and monitoring cardiovascular changes occurring during laparoscopic cholecystectomy.

## Background

It has been proven by previous studies that inducing a positive pressure within the intraperitoneal cavity during laparoscopic surgical interventions results in numerous cardiovascular, neuroendocrine and renal changes [1]. These changes include an increase in systemic and pulmonary vascular resistance and a consequent decrease in cardiac output, which may be attributed to direct mechanical factors due to intraperitoneal pressure rise as well as to humoral changes evoked by the procedure [2].

During preoperative anesthesiological consultation it is a frequent question whether laparoscopic cholecystectomy can be performed safely in patients with known cardiovascular risk factors. It is worth mentioning that in a study of low risk patients undergoing laparoscopic cholecystectomy, 2 out of 16 patients had acute ST changes on their ECG [3]. In view of this, preoperative cardiovascular risk stratification as well as proper intraoperative monitoring may be of high importance in patients at risk.

So far, cardiovascular consequences occurring during laparoscopic procedures have been assessed either during animal experiments [4] or by using invasive intraoperative hemodynamic monitoring of humans [5–9]. However, invasive hemodynamic monitoring techniques may also have side effects and thus are not indicated in all patients undergoing otherwise relatively short and low risk surgical procedures.

Along these lines, we tested the hypothesis that cardiovascular changes caused by CO_2_-pneumoperitoneum may be accurately assessed intraoperatively by non-invasive applanation tonometry. Our results were compared with data obtained from the literature.

## Patients and methods

### Patients

A total of 41 consecutive patients undergoing elective laparoscopic cholecystectomy for symptomatic cholelithiasis without cholangiography or choledochotomy were enrolled in this prospective case-series. The patients were all in good health, classified as ASA I and II. This study was conducted with approval from the University of Debrecen Medical Ethics Committee (Registration number: DEOEC RKET/IKET 2312-2010, responsible person: József Szentmiklósi; Department of Pharmacology, University of Debrecen, 98.Nagyerdei krt. Debrecen, Hungary, Phone: +3652411600). A written informed consent to participate was obtained from all patients included in the study. Patients with diabetes mellitus, untreated hypertension, atrial fibrillation, morbid obesity (body mass index [BMI] > 35), infection, psychiatric or neurologic conditions impairing patients’ ability to cooperate were excluded from the study.

### Anesthesia and CO_2_ pneumoperitoneum

General anesthesia was administered to all patients according to the same protocol. During the patient’s stay in the preparation area, 15 ml/kg/BW of Ringer’s acetate solution was infused for a period of 2 hours. As premedication, oral midazolam (0.15 mg/kgBW) was administered 30 minutes before the induction of anaesthesia. Following preoxygenation (2 minutes) by face mask, anesthesia was induced with intravenous propofol (2 mg/kg BW) and fentanyl (3-5 μg/kgBW). Rocuronium 0.6 mg/kgBW was used to facilitate tracheal intubation and maintain muscle relaxation. After intubation, the lungs were ventilated with a mixture of air/oxygen (50/50%). For maintenance of anesthesia sevoflurane (2 vol%) and intermittent doses of fentanyl were applied. Sevoflurane was titrated in order to keep the bispectral index (BIS) values between 40 and 50. Ventilation was mechanically controlled at a frequency and tidal volume sufficient for maintaining normocapnia. PEEP was not administered (ZEEP). End expiratory CO_2_ was used to ensure normoventilation (end-tidal carbon dioxide level was kept between 35 and 38 mmHg). Intraoperative crystalloid infusion was administered at 7 mL/kg per hour. During anesthesia body temperature was maintained between 36,0 and 36,5°C by heating blankets.

The surgical technique was similar for all patients. CO_2_ intraperitoneal pressure was maintained automatically at a recommended 12-14 mmHg by a CO_2_ insufflator at an insufflation rate of 1 to 1,5 L/min with the patients placed in the 20° reverse Trendelenburg position (rT). On confirming the appropriate placement of the video laparoscope, each patient’s position was changed to a left lateral tilt (10°-15°). Once the surgery was completed, the abdomen was deflated and each patient was returned to the horizontal position.

Routine intraoperative patient monitoring included continuous five-lead electrocardiography, pulse oximetry, non-invasive blood pressure measurements, peak airway pressures, capnography, as well as BIS monitoring for assessment of depth of anesthesia. Neuromuscular monitoring was performed to control the neuromuscular block throughout the course of anesthesia using TOF Watch SX acceleromyograph.

### Monitoring cardiovascular function

The SphygmoCor pulse wave analysing system was used for monitoring cardiovascular function, which is a non-invasive method based on applanation tonometry [10]. During the present study we measured systemic and central aortic pressure, augmentation pressure, augmentation index, ejection duration and subendocardial viability ratio.

- *Measurement of central aortic pressure and aortic pressure waveform:* A conventional cuff pressure measurement was used for calibration. After applanation tonometry SphygmoCor derived a complete waveform for the whole cardiac cycle for the aortic pulse. A combination of the two methods makes it possible to analyse the coupling between the ejecting heart and the pressure load.
- *Measurement of augmentation pressure and augmentation index:* Augmentation pressure is based on the principle that there is a reflected pressure from the periphery that appears in the aortic pressure waveform. The amount of augmentation reflects the stiffness of the peripheral arterial tree: it increases along with higher stiffness. In order to make the value of augmentation index independent from the individual changes of pulse rate, the device calculates a corrected augmentation index (ALX@HR75).
- *Subendocardial viability ratio:* This parameter is calculated by the device by dividing the area under the systolic and diastolic part of the curve. A ratio under 100% reflects underperfusion of the subendocardium.

Hemodynamic measurements were repeated in different phases of the procedure: Before induction of anesthesia (resting phase, Time 1); 5 minutes after induction of anesthesia (Time 2.); 5 minutes after inflation of the peritoneal space (Time 3.) followed by repeated measurements every 10 minutes and 5 minutes after deflation of the peritoneal cavity (Time 4).

### Statistical analysis

Means and standard deviation were calculated for all values. Repeated measure analysis of variance was used for all values to check the time main effect of the laparoscopic procedure, i.e. whether laparoscopic cholecystectomy overall had any significant hemodynamic effect. Pairwise comparisons of all parameters were performed in order to check the effect of inflation and deflation of the peritoneal space by taking the values obtained after induction of anesthesia as reference value. A p < 0.05 was considered as a statistically significant difference.

## Results

A total of 41 patients entered the study. There were 33 females and 8 males with an average age of 52.3 ± 15.4 years.

### The effect of anesthetic induction on hemodynamic parameters

As shown in Table 1, induction of anesthesia resulted in a statistically significant reduction in both peripheral blood pressure and central aortic pressures, accompanied by a decrease in augmentation pressure and augmentation index.

**Table 1:** **The effect of anesthetic induction on peripheral and central (aortic) blood pressures, ejection duration and pressure augmentation**

	Before induction	After induction	p-value
**Peripheral blood pressure**			
**Systolic**	132.47 ± 18.87	116.80 ± 18.61	P < 0.001
**Diastolic**	78.60 ± 10.40	72.71 ± 13.54	0.01
**Pulse pressure**	52.12 ± 16.12	43.65 ± 11.96	P < 0.01
**Central (aortic) blood pressure**			
**Systolic**	120.80 ± 19.10	106.77 ± 18.78	P < 0.001
**Diastolic**	80.00 ± 10.38	73.88 ± 13.79	P < 0.01
**Pulse pressure**	41.05 ± 14.70	33.11 ± 11.08	P < 0.001
**Ejection duration**	41.0 ± 4.93	38.40 ± 6.71	0.01
**Pressure augmentation**			
**Augmentation pressure**	10.52 ± 8.52	7.31 ± 5.59	P < 0.01
**Augmentation index**	23.62 ± 10.58	18.97 ± 10.80	P < 0.01
**Subendocardial viability ratio (%)**	121.85 ± 22.7	142.5 ± 38.2	P < 0.01

Means and standard deviations are shown.

### The effect of peritoneal insufflation on hemodynamic parameters

Table 2 summarizes the parameters that were obtained before and after peritoneal cavity insufflation. Peripheral blood pressures did not change markedly along with peritoneal cavity insufflation, except for a moderate increase in systolic blood pressure. In contrast to this, a marked increase could be observed in central aortic pressure values which was accompanied by increased augementation pressure and augmentation index, indicating a rise in peripheral arterial stiffness. Despite changes in the central aortic blood pressure, subendocardial viability ratio remained relatively stable during and after peritoneal cavity insufflation.

**Table 2:** **The effect of peritoneal cavity insufflation on peripheral and central (aortic) blood pressures, ejection duration and pressure augmentation**

	Before insufflation	After insufflation	p-value
**Peripheral blood pressure**			
**Systolic**	116.80 ± 18.61	125.17 ± 20.21	0.02
**Diastolic**	72.71 ± 13.54	78.92 ± 16.94	0.07
**Pulse pressure**	43.65 ± 11.96	44.64 ± 13.23	0.24
**Central (aortic) blood pressure**			
**Systolic**	106.77 ± 18.78	118.05 ± 19.85	P < 0.01
**Diastolic**	73.88 ± 13.79	81.82 ± 12.28	P < 0.01
**Pulse pressure**	33.11 ± 11.08	36.41 ± 12.60	0.04
**Ejection duration**	38.40 ± 6.71	39.15 ± 5.51	0.34
**Pressure augmentation**			
**Augmentation pressure**	7.31 ± 5,59	12.61 ± 7.56	P < 0.001
**Augmentation index**	18.97 ± 10.80	31.55 ± 12.01	P < 0.001
**Subendocardial viability ratio (%)**	142.5 ± 38.2	137.11 ± 26.3	P = 0.48

Means and standard deviations are shown.

### Comparison of hemodynamic parameters at preeinduction and peritoneal insufflation phase

Peripheral and central blood pressures returned to the preinduction values after peritoneal insufflation (comparison of Phase 1 and 3). The only parameter that showed a gradual increase was augmentation index (Table 3).

**Table 3:** **Comparison of peripheral and central (aortic) blood pressures, ejection duration and pressure augmentation values at phases before induction and after peritoneal insufflation**

	Before induction	After insufflation	p-value
**Peripheral blood pressure**			
**Systolic**	132.47 ± 18.87	125.17 ± 20.21	0.10
**Diastolic**	78.60 ± 10.40	78.92 ± 16.94	0.91
**Pulse pressure**	52.12 ± 16.12	44.64 ± 13.23	0.02
**Central (aortic) blood pressure**			
**Systolic**	120.80 ± 19.10	118.05 ± 19.85	0.53
**Diastolic**	80.00 ± 10.38	81.82 ± 12.28	0.47
**Pulse pressure**	41.05 ± 14.70	36.41 ± 12.60	0.13
**Ejection duration**	41.0 ± 4.93	39.15 ± 5.51	0.11
**Pressure augmentation**			
**Augmentation pressure**	10.52 ± 8.52	12.61 ± 7.56	0.25
**Augmentation index**	23.62 ± 10.58	31.55 ± 12.01	<0.01
**Subendocardial viability ratio (%)**	121.85 ± 22.7	137.11 ± 26.3	<0.01

Means and standard deviations are shown.

**Figure 1:**
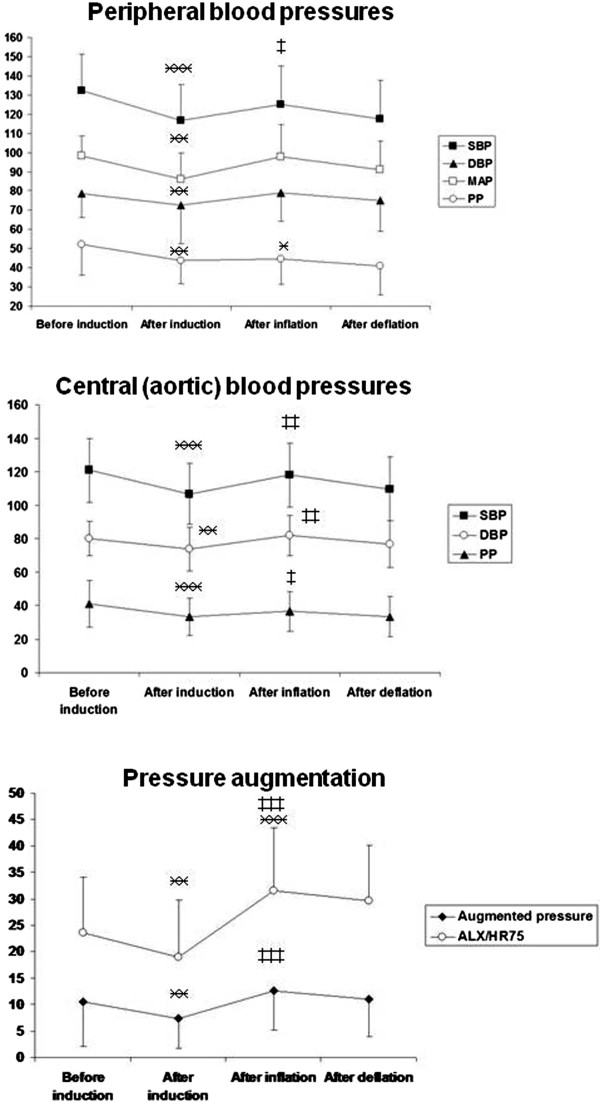
**Changes of hemodynamic parameters during the study: Means and standard deviations are shown.** ӿ ӿ indicate p < 0.01, ӿ ӿ ӿ indicate p < 0.001 compared to preinduction value; ‡ ‡ p < 0.01, ‡ ‡ indicate p < 0.001 statistical difference compared to preinsufflation value.

*After deflation of the abdominal cavity* both peripheral and aortic pressure values returned to the levels observed after induction of anesthesia. Although augmentation pressures were still higher than before inflation, augmentation index (the main indicator of peripheral arterial stiffness) also returned to the pre-insufflation value. Figure 1 depicts and summarizes the changes of all parameters during the entire course of the study.

## Discussion

In this cohort study we used a new non-invasive technique for assessing the cardiovascular changes that occur during laparoscopic cholecystectomy, the Sphigmocor pulse wave analysis. The method has already been tested in different clinical conditions such as arterial hypertension, diabetes mellitus, systolic heart failure and preeclampsia [11–13]. In anesthesiological practice, this is the first report on the use of the technique.

Similar to previous reports we were able to detect a significant decline of the systemic blood pressure after anesthetic induction which was accompanied by a decrease in augmentation index, reflecting the stiffness of the peripheral vessels [3, 5, 9, 14, 15]. This initial reduction in blood pressure and peripheral resistance may be due to the direct myocardial depressant and vasodilatory effects of the anesthetics together with the loss of sympathetic tone [2]. During the next phase of the procedure, after inflating the abdomen and tilting the patient to a reverse Trendelenburg position, the most important observations were increases in central aortic pressures accompanied by an increase of augmentation index. This is in line with previous observations reporting on an increase in mean arterial pressure and peripheral resistance [1, 2, 5, 9, 14] after peritoneal insufflation. To transform these results to our observation, we have to mention that systolic central aortic pressure increased by 10,6%, whereas augmentation index referring to peripheral resistance increased by 66% on average after peritoneal insufflation. It has to be noted that hemodynamic parameters in this phase returned to the baseline, preinduction values (Table 3). It seems that the effect of inducing pneumoperitoneum counteracts the hemodynamic depressant effects of the anesthetics suggesting that changes in ASA I-II patients are clically most probably not relevant. In a recent study, Cinnella and co-workers also demonstrated that hemodynamic stability after administering pneumoperitoneum is maintained even if moderate (5 cm H_2_O) is applied [16].

In a previous review Wahba an co-workers [2] summarized the hemodynamic effects and suggested that direct mechanical, neurohumoral processes play a role, slightly modified by the effect of the resorbed CO_2_ during pneumoperitoneum. Mechanical effects of pneumoperitoneum may decrease renal flow, activating the renin-angiotensine-aldosteron system, may result in the compression of the abdominal veins and the aorta. It has also been proven that inducing pneumoperitoneum leads to an increased production of vasopressin, adrenalin, noradrenalin, renin and cortisol, which is in correlation with the changes of mean arterial pressure and systremic resistance [15]. According to previous reports, this is the phase of laparoscopic cholecytectomy where patients of different ASA severity (ASA I-II vs. ASA III-IV) may react differently to pneumoperitoneum. In more severe patients (ASA III-IV) a pressure rise in the abdomen resulted in a more pronounced increase in mean arterial pressure and decreased oxygen delivery [17]. Consequently, left ventricular stroke work index increases, which causes higher oxygen demand of the myocardium [7]. In our series we included ASA I and II patients and no subendocardial viability ratio reflecting the potential underperfusion of the subendocardium could be detected after and during the course of abdominal inflation.

The principal basis of the pulse wave analysis system is that the peripheral arterial pressure waveform may be used for the reconstruction of central (aortic) pressure. The method behind this is applanation tonometry, which ensures the sensitive detection of the radial artery pulse waveform. It is generally accepted that the characteristics of the peripheral pulse reflect the changes in arterial diameters, wall elasticity, wall thickness and the condition of the peripheral vascular beds. The main attribute of SphygmoCor is its ability to derive the central aortic pressure waveform non-invasively from the pressure pulse recorded at a peripheral site, usually at the upper arm (radial artery) [10].

### Limitations

The intraoperative use of the device is limited by the position of the radial artery, i.e. in some surgical scenarios it may disturb the surgical team, making monitoring impossible. Another limitation to be mentioned is operator-dependency: for reliable monitoring it is necessary to have previous experience with the technique. Finally, the main limitation of this study is the lack of a control group, i.e. other hemodynamic measurements were not used. However, this trial is a pilot application of applanation tonometry in this field. As this is a non-invasive method, in this first, pilot step of our investigations we intended to compare hemodynamic changes with those that used invasive monitoring techniques reported in the literature.

## Conclusion

In conclusion: in this study we have shown that the Sphigmocor pulse wave analysis system can be reliably used for detecting and monitoring cardiovascular changes occurring during laparoscopic cholecystectomy. Further studies are needed to prove whether the method may be helpful in delineating critical situations in patients with limited cardiovascular reserve (ASA III-IV patients) by defining cut-off values of safety and to help in guiding abdominal insufflation and tilting during the procedure as suggested in previous reports [18].
